# Reducing occupational sitting: Workers’ perspectives on participation in a multi-component intervention

**DOI:** 10.1186/s12966-017-0530-y

**Published:** 2017-05-30

**Authors:** Nyssa T. Hadgraft, Lisa Willenberg, Anthony D. LaMontagne, Keti Malkoski, David W Dunstan, Genevieve N Healy, Marj Moodie, Elizabeth G Eakin, Neville Owen, Sheleigh P Lawler

**Affiliations:** 1 Physical Activity Laboratory, Baker Heart and Diabetes Institute, Melbourne, VIC Australia; 2 School of Public Health and Preventive Medicine, Monash University, Melbourne, VIC Australia; 3 Centre for International Health, Burnet Institute, Melbourne, VIC Australia; 4 Centre for Population Health Research, Deakin University, Melbourne, VIC Australia; 5 Schiavello International, Melbourne, VIC Australia; 6 Mary MacKillop Institute for Health Research, Australian Catholic University, Melbourne, VIC Australia; 7 School of Exercise and Nutrition Sciences, Deakin University, Burwood, VIC Australia; 8 School of Sport Science, Exercise and Health, The University of Western Australia, Perth, WA Australia; 9 Department of Medicine, Monash University, Melbourne, VIC Australia; 10 The University of Queensland, School of Public Health, Brisbane, QLD Australia; 11 School of Physiotherapy and Exercise Science, Curtin University, Perth, WA Australia; 12 Swinburne University of Technology, Melbourne, VIC Australia; 13 Melbourne School of Population & Global Health, The University of Melbourne, Melbourne, VIC Australia

**Keywords:** Workplace, Sedentary behaviour, Sitting, Intervention, Qualitative

## Abstract

**Background:**

Office workers spend much of their time sitting, which is now understood to be a risk factor for several chronic diseases. This qualitative study examined participants’ perspectives following their involvement in a cluster randomised controlled trial of a multi-component intervention targeting prolonged workplace sitting (Stand Up Victoria). The intervention incorporated a sit-stand workstation, individual health coaching and organisational support strategies. The aim of the study was to explore the acceptability of the intervention, barriers and facilitators to reducing workplace sitting, and perceived effects of the intervention on workplace culture, productivity and health-related outcomes.

**Methods:**

Semi-structured interviews (*n* = 21 participants) and two focus groups (*n* = 7) were conducted with intervention participants at the conclusion of the 12 month trial and thematic analysis was used to analyse the data. Questions covered intervention acceptability, overall impact, barriers and facilitators to reducing workplace sitting, and perceived impact on productivity and workplace culture.

**Results:**

Overall, participants had positive intervention experiences, perceiving that reductions in workplace sitting were associated with improved health and well-being with limited negative impact on work performance. While sit-stand workstations appeared to be the primary drivers of change, workstation design and limited suitability of standing for some job tasks and situations were perceived as barriers to their use. Social support from team leaders and other participants was perceived to facilitate behavioural changes and a shift in norms towards increased acceptance of standing in the workplace.

**Conclusions:**

Multi-component interventions to reduce workplace sitting, incorporating sit-stand workstations, are acceptable and feasible; however, supportive social and environmental conditions are required to support participant engagement. Best practice approaches to reduce workplace sitting should address the multiple levels of influence on behaviour, including factors that may act as barriers to behavioural change.

**Electronic supplementary material:**

The online version of this article (doi:10.1186/s12966-017-0530-y) contains supplementary material, which is available to authorized users.

## Background

The modern office workplace is conducive to workers spending large amounts of time sitting [1], which is a potential risk factor for chronic disease and premature mortality [2–4]. In recent years there has been increasing interest in understanding the efficacy of a broad range of interventions targeting workplace sitting [5]. Sit-stand workstations, which facilitate postural shifts from sitting to standing and vice versa throughout the day, have been shown to be an effective environmental-based tool, leading to moderate to large reductions in sitting time [6, 7] with minimal or no impacts on productivity or work performance [8–10]. However, evidence reviews have suggested that greater impacts on workplace sitting may be achieved if sit-stand workstations are implemented as part of a broader approach that addresses the multiple levels of influence on behaviour [5, 11, 12]. While previous qualitative studies have assessed workers’ perspectives of sit-stand workstations in isolation [13–16], there is limited knowledge about the feasibility and acceptability of incorporating them within a multi-component, participatory workplace intervention.

The recently completed Stand Up Victoria (SUV) trial demonstrated that a multi-component approach, incorporating individual, organisational and environmental-level strategies, was effective at reducing both total and prolonged workplace sitting time relative to a control group at three and 12 month follow-up [17]. As one of the first multi-component workplace interventions targeting workplace sitting, understanding the participant perspective can help to identify the factors that contributed to its effectiveness.

Previous qualitative research has generally evaluated perceptions of sit-stand workstations in isolation, trialled over a short time period (1 month or less) [13, 16]. These findings suggest that sit-stand workstations are considered to be acceptable and feasible, although issues associated with the design of certain models, and concerns about reduced audio and visual privacy with standing have been raised as potential barriers to their use [13, 15, 16]. There is a need to understand the longer-term feasibility and sustainability of a multi-component workplace intervention featuring sit-stand workstations, including the role of broader workplace culture and organisational factors in supporting reductions in sitting time. The present study examined participants’ perspectives of a multi-component intervention to reduce workplace sitting, including the acceptability of the intervention, barriers and facilitators to reducing workplace sitting, and perceived effects on workplace culture, productivity and health-related outcomes.

## Methods

### Study setting and design

This qualitative study was part of the broader Stand Up Victoria (SUV) trial. SUV, conducted in Victoria, Australia, was a 12 month cluster-randomised controlled trial of a multi-component workplace intervention to reduce prolonged sitting in office workers. Full details of the study design [18], intervention development [19], and main outcomes [17] have been described previously. In brief, participants were recruited between 2012 and 2013 from selected teams at 14 different worksites of a government department. Randomisation to control or intervention condition (seven sites for each) occurred at the worksite-level. The SUV trial was granted approval by the Alfred Health Human Ethics Committee (Melbourne, Australia) and had prospective trial registration with the Australian New Zealand Clinical Trials register (ACTRN12611000742976; registered 15 July 2011). Participants provided written informed consent.

### Intervention

The intervention was multi-component and comprised organisational-, environmental [sit-stand workstations]-, and individual-level strategies (see [18] for further details), with the primary aim being to reduce workplace sitting time. Strategies included an individual sit-stand workstation (Ergotron WorkFit-S; www.ergotron.com) that was retained by each participating worker for 12 months; face-to-face and telephone health coaching (for the first 3 months); and organisational-level strategies that were selected through a group participatory brainstorming session at each worksite prior to commencement of the intervention. Ongoing organisational support was provided by team champions (typically team leaders) who promoted the selected strategies and the intervention messages of “Stand Up, Sit Less, Move More”. In general, workstations were removed at the end of the 12 month trial, however some participants retained them for medical reasons.

### Procedure

At the final 12 month assessment, intervention participants were asked whether they wished to be contacted about contributing to further research. Following completion of the trial, those who opted in (*n* = 56 of 94 who completed the online questionnaire) were contacted by telephone and offered the opportunity to partake in either a face-to-face interview at their workplace, or a telephone interview at a time that was convenient for them. The option of participating in a focus group discussion was offered to participants at one of the intervention sites due to a high proportion of intervention participants opting in for an interview. Team leaders from six of the seven intervention sites consented to participate and were able to provide managerial/supervisory perspectives of the implementation of the intervention within their teams. The number of participants at each site ranged from two to five. Five interviews were conducted face-to-face at participants’ workplaces and 16 were conducted by telephone (*n* = 21). Two focus groups were conducted, involving three and four participants, respectively (*n* = 7). Interviews and focus groups occurred across the seven intervention sites between July 2013 and December 2014 (between one and 4 months after participants completed the final questionnaire).

Each interview was semi-structured and conducted by one of the authors (LW) using an interview guide. This researcher had postgraduate qualifications in public health and previous experience conducting qualitative interviews. At the time of the interviews, she was employed as the SUV project coordinator and was known to the participants through this position, and her role in conducting the onsite assessments. The interview guide was adapted for the focus groups, to be appropriate for the needs of a group discussion (Additional file 1). Both focus group sessions were facilitated by a member of the research team (LW), ran for approximately 45 min to 1 h, and were digitally audio-recorded. Interview/focus group question topics covered the feasibility and acceptability of the individual, organisational and environmental components of the intervention; perceived productivity and health effects of reducing workplace sitting time; and organisational support for the key messages of the study (Table 1). Participating team leaders were also asked about the role they played as “champions” throughout the intervention duration. Participants were advised that the researchers had no commercial interest in the workstations, and were therefore encouraged to speak freely about this aspect of the intervention.

**Table 1:** Questions covered during the interviews for employees and team leaders

Theme: Global Satisfaction
1. How was your overall experience with the Stand Up Victoria study? (All participants)
• How satisfied were you with your experience? • What were some of the positives about the experience? • What do you see as the physical advantage/s of standing more at work? • What do you see as the cultural advantage/s of standing more at work? • What do you see as the disadvantages of standing more at work? Are there any areas for development/improvement? • Do you feel that the movement improved your wellbeing/comfort? How? • What could be improved upon in future projects that are introducing sit/stand workstations? (Be sure to let participants know that we have no commercial interest in the workstations) • Did you feel that you/your team were provided with the right knowledge to allow you to stand up, sit less, and move more within your workplace? • Would you recommend sit-stand works stations to other teams/workplaces? Why/Why not?
Theme: Motivation and sustainability
Interviewer to bring in the strategies that they agreed on at the initial group info session
2. Thinking about your team as a whole... (Team leaders)
• What strategies worked? Why do you think that is? • What did you see as the most commonly used strategy by your team? (If stated above, skip this question) • Are your team still using these strategies? • If changes were made, what is it going to take for these changes to become sustainable in your group in the long-term? • What didn’t work? Why do you think that they didn’t work? • Did you feel that your team had enough knowledge of the product to make the changes to their working position? • How suitable do you think your workplace is for sit/stand workstations? If not, what needs to change to make it suitable? • Could you see your workplace taking on any other changes now that the study is complete?
3. If you could reflect on your own individual strategies... (Employees)
• Which ones motivated you the most to change your working position? Why do you think that was? • Where there any strategies that you tried that didn’t work? Why do you think that was? • If changes were made, what is it going to take for these changes to become sustainable in your group in the long-term? • Now that the study is over and your desks have been removed, are you still trying to follow the objectives of the study – standing up, sitting less, moving more? • How suitable do you think your workplace is for sit/stand workstations? If not, what needs to change to make it suitable? • Could you see your workplace taking on any other changes now that the study is complete?
Theme: Workplace Culture
4. To what extent do you feel the workplace ‘culture’ has changed to support the Stand Up Sit Less Move More messages? (All participants)
• Did you feel you had the support of senior/upper management to make these changes within your team? Why, why not? (Team leaders only) • Did you feel you had the support of your team leader/upper management to make these changes at your workstation? Why, why not? (Employees only) • Was it an accepted norm to stand and use the workstation? • Did the opportunity to have a sit-stand workstation make you feel more valued as employees? (Employees only) • Did the sit-stand workstation make you feel more in control of your workspace? (Employees only)
5. Did you feel that the sit-stand workstation impacted on your/your team’s sense of privacy – either audio or visual privacy? (All participants)
• Did you feel that the sit-stand workstations impacted on the sense of visual privacy of others around you? If yes, how so? • Did you feel that the sit-stand workstations impacted on the sense of audio privacy of others around you? If yes, how so?
6. Did managers let non-participants know about the study and the changes that were going to be made to the workplace by having the workstations installed? (Team leaders)
• Did you witness any informal or formal negotiation between participants and non-participants with respect to the utilisation of the workstations? • Was there any feedback as a result of these changes to the workplace? • If any, were they mainly from participants or non-participants? • Did the increased standing infiltrate to other non-participant team members? I.e., was there a ripple effect or by-product of having others standing within your workplace? • Did you feel that the level of movement in general changed around the workplace during the study period? If there was a change, what impact did this have on the way in which your team worked? (positive/negative?)
Theme: Empowerment (Team leaders only)
7. As a team leader, did you feel you had a responsibility to act as a role model for the duration of the study? If so, how did you find this leadership role?
• Do you feel that your efforts were recognised? (by management/other staff) • Was it a positive responsibility/role to have? If not, why? • Do you feel that your leadership role made a contribution to any changes that occurred in your work place? If so, how? If not, why?
Theme: Productivity
8. What did you think about the impact of the workstations on you/your team’s productivity, in terms of: (All participants)
• Communication (between each other and clients) • Collaboration • Timeliness of task completion/ work flow

To assess whether additional themes arose that were not anticipated during the development of the original interview guide, the first 11 interviews were transcribed and examined. Through this process, unanticipated themes relating to the impact of the intervention on non-participants were identified. Subsequently, additional prompts were added to the interview guide for the remaining interviews, to further explore these new findings. Interviews ran for approximately 20–40 min and were all digitally audio-recorded. All participants provided verbal consent at the beginning of the interviews and focus groups for the audio recording.

### Analysis

The audio-recordings were transcribed verbatim. For one interview, only a partial recording was available for analysis due to technical issues. Initial analysis of the data was conducted by two of the authors using Microsoft Word (LW) and NVivo 11 (QSR International Pty Ltd.) (NH) software, separately for the interview and focus group data. A familiarisation process was conducted first by reading and re-reading each transcript. Each researcher then independently coded each transcript, with codes identified based on a priori themes of interest and emergent themes. Initial codes were then grouped together into sub-themes and overarching themes and relevant data to each theme collated. The coding frameworks developed by the two researchers were then compared to identify similarities and discrepancies. A third researcher (SL) coded a subset of the interviews and was involved in discussions around the final themes. This process enabled resolution of any differences between the two initial coders and led to consensus on the names and descriptions of the final themes and sub-themes. Quotes from participant interviews were selected to portray the content of each theme/sub-theme.

## Results

### Participants

Participant baseline characteristics for this study, as well as the baseline characteristics of the whole intervention group are described in Table 2. Participants were broadly similar to all SUV participants in the intervention arm of the trial in terms of socio-demographic and work-related characteristics, but had higher reductions in workplace sitting. Similar themes emerged from the interview and focus group data, although the focus groups revealing richer data relating to workplace culture and team dynamics. For simplicity of reporting, data from the interviews and focus groups were combined.

**Table 2:** Participant characteristics at baseline

	Interview participants	Focus group participants	All intervention participants
n	21	7	136
Gender (women)	12 (57%)	6 (86%)	89 (65%)
Age (years)	48.9 (8.5)	45.6 (11.3)	44.6 (9.2)
% Married/living together	15 (71%)	2 (29%)	86 (64%)^a^
BMI (kg/m^2^)	27.9 (4.0)	24.6 (4.4)	28.6 (6.5)
% Tenure >5 years	16 (76%)	5 (71%)	94 (70%)^a^
% 1.0 FTE	16 (76%)	6 (86%)	107 (80%)^a^
Mean change in workplace sitting (mins) baseline-12 months^b^	−97.9 (121.4)	−89.5 (125.3)	−78.8 (100.6)^c^

n (%) or mean (SD). *FTE* full-time equivalent, *BMI* Body mass index

^a^n = 134

^b^Unadjusted data

^c^97

Themes and illustrative quotes are listed below. These are grouped into the following key areas: overall experience of the intervention; work performance and productivity; organisational support and workplace culture; and processes of behavioural change. An overview summary of the number of participants responding to each theme is provided in Table 3.

**Table 3:** Summary of participants’ responses to each theme

Themes	Interviews (*n* = 21)	Focus groups (*n* = 7)
	n	n
Overall experience^a^	Positive: 21	Positive: 7
	Negative: 4^a^	
Awareness raising	Positive: 15	Positive: 4
	Neutral: 1	
Improved health and well-being^a^	Positive: 18	Positive: 5
	Negative: 3	
Work performance and productivity		
Effects on work performance	No effect: 11	Positive: 4
	Positive: 5	
	Negative: 2	
Long-term productivity outcomes	Positive: 2	Positive: 2
Workstation design^a^	Negative: 12	Negative: 7
	Positive: 1	
	Neutral: 6	
Communication and team dynamics^a^	Positive: 9	Positive: 3
	Negative: 1	Negative: 1
	Neutral: 2	
Organisational support and workplace culture		
The importance of social support^a^	Positive: 16	Positive: 5
	Negative: 1	Negative: 1
	Neutral: 1	
Intervention effects on non-participants^a^	Positive: 4	Positive: 2
	Negative: 5	Negative: 3
	No effect: 6	No effect: 3
Organisational support post-intervention^a^	Uncertain: 8	Uncertain: 2
	Certain (for OHS issue/request): 9	Certain (for OHS issue/request): 4
Processes of behavioural change		
Sit-stand workstations as the key facilitator of behavioural change^a^	Yes: 13	Yes: 7
	No: 1	
Diversity in use and engagement with intervention strategies around ‘stand up, sit less and move more’	Yes: 21	Yes: 7
Health coaching and behavioural change	Useful: 13	Useful: 4
	Neutral/not useful: 6	Neutral/not useful: 3

^a^Note: some participants in more than one category

### Overall experience

Participants’ overall experience of the SUV intervention was very positive, with participants enjoying the opportunity provided by participating in the study. While a small number reported a negative component of their experience, these participants also additionally reported positive aspects of participation (see Table 3).

*I thought it was a really good, unique kind of experience. I have worked for (organisation) for 11, 12 years and this is the first time I have been involved in such a unique initiative.* Site J, Participant 8 (J8), male team leader

#### Awareness raising

Participants reported that the SUV intervention increased awareness of their own behaviour—particularly how much time they spent sitting at work—and the health consequences of excessive levels of sitting.

*You didn’t really think about it that much but now that I have been forced to stand up you start taking your health more seriously. And just think… maybe I shouldn’t be sitting down for so long.* L10, female employee

*It’s changed my whole mindset, even at home, not just at work. I’m constantly now aware of sitting for more than 30 minutes at a time. I never was aware of that before.* H3, female employee

The reports provided after each assessment summarising the objective data from the activity monitors were particularly important in facilitating this awareness raising and helped participants to understand how sedentary they were in the workplace.

*Seeing it on paper – seeing my graph, I think that was a really positive part of the trial – that was very impacting on me, to visually see what I did for a week before I actually went on the trial and then see, “Oh my God, I sat that much.”* H3 female employee

For some participants the intervention had a broader impact on awareness beyond workplace sitting. It was an *“awakening”* that prompted them to think about their health more generally.

*It’s definitely helped to highlight that I need to look after myself more. I need to drink more water, I need to be moving so that I'm not, yeah, stagnant and, yeah, bringing on any* [health] *conditions I guess.* M8, female employee

Many participants noted that since the study concluded, they found it difficult to sit for prolonged periods of time; one participant noted that they now started to feel *“edgy”* (C9, female employee). However, with the desks removed it became more difficult to break up their sitting time.

#### Improved health and well-being

The intervention was generally considered to have had a positive impact on both physical and mental well-being. Some participants reported that replacing some sitting with standing alleviated musculoskeletal issues (such as neck or back pain) or that physically they generally felt better. Participants also reported feeling more alert, having greater concentration and energy as a result of increased time spent standing.

*Before I started the study I was experiencing some pain in my shoulder and neck on the left side, and I found that by standing it actually alleviated those problems.* M9, female employee

*When you're standing up you felt so much looser, I suppose. After sitting down for a while you got really stodgy and sluggish, but when you're standing up you feel a lot freer, more relaxed, more alert. You concentrate better, I suppose.* K4, male employee

However, when the desks were removed at the end of the intervention participants noted musculoskeletal pain returning.

*After going through hoops I managed to keep the desk but there was a period of time for about six weeks where I didn’t have the desk and I immediately got back pain. I didn’t tell anybody anything because I didn’t want to come across like I was just whinging but I actually went home with really sore back pain through that four to six weeks where I didn’t have the stand up desk.* H3, female employee

### Work performance and productivity

#### Effects on work performance

Participants generally did not believe that the use of the sit-stand workstations had any impact on their productivity, either positive or negative. Some thought that it may have had a positive impact on their work performance as a result of perceived improvements in alertness and concentration (see Table 3).

*Having the stand up desk definitely helps me to communicate with my customers that I’m speaking to. It helps me to think. I’m much more productive with a stand up desk. I’m clear minded, I’m focussed, I’m standing, I’m getting oxygen in me.* H3, female employee

However, work tasks sometimes made it difficult for participants to use their workstations in the standing position. Some reported that they chose to sit for certain tasks to increase their audio and visual privacy, for example, when taking more complex phone calls with clients or when dealing with sensitive information on their computers.

*In difficult clients, I had to sit down even though, if I had my freedom, I probably would be standing up because I just needed to be able to have a close conversation with the client without the background noise.* J7, female employee

#### Long-term productivity outcomes

A couple of participants remarked that the provision of sit-stand workstations had the potential to lead to productivity benefits for the organisation in the longer-term as a result of reductions in absenteeism and potentially avoiding compensation claims.

*It’s also about getting people back to work as well too – [one of the girls in our team’s] got a back injury and her physician basically gave her a five day pass to not come to work because of her back, but because she had the stand-up, sit-down desk she said, “I was able to come to work because I could stand and relieve that pressure” – so there’s five days there of a person who wouldn’t have come to work.* FG2 H24, female employee

#### Workstation design

While the concept of having a sit-stand workstation was appreciated and valued, nearly all participants reported issues with the design of the model (*Ergotron WorkFit-S;* www.ergotron.com), with some reporting that this impacted on their work performance. Most considered the size of the platforms to be too small to accommodate the mouse and for work tasks requiring hard copy documents. Some reverted back to sitting so that they could access the larger work surface on their normal seated desk. Others expressed dissatisfaction with the workstation stability, noting that the platform shifted too easily from standing to sitting with minimal force applied.

*In my role that I do…I write a lot. So there is not enough adequate space for me to actually write properly and feel comfortable at the desks, so I suppose I sat a lot. In the beginning I was standing up but even when you are standing up or sitting down you have only got that tiny little area around you.* L18, female employee

#### Communication and team dynamics

There was a perception that increased standing facilitated communication with co-workers and team members, as participants were more visible in the open plan office. For team leaders this was considered positive, whereas other employees found it distracting when it interrupted their work flow.

*I felt like I was more connected to my team because I could see more, I could instantly – not instantly run around, but you know I was a bit more hands-on, rather than when you’re sitting you sort of wait until something’s got your attention.* FG1 H6, female team leader

*I think it actually improved interaction with colleagues because you’re sitting at your desk you see the tops of people’s heads but you never actually talk to people, but you stand and you’re actually making eye contact with people and having a wave… if you’re sitting at your desk you just never see anybody, I mean people are right beside you and it’s almost as if they don’t exist.* FG2 H19, female employee

### Organisational support and workplace culture

#### The importance of social support

The collegiality and peer support experienced by sharing the intervention experience with co-workers was valued and appeared to encourage participants to increase their standing and movement. As indicated in Table 3, the social support from other participants and team leaders was perceived by the majority of participants to have been an important facilitator of behaviour change and engagement with intervention strategies. In particular, participants reported using others’ behaviour as a prompt to remind them to stand up.

*I was lucky because I had two other people in my row that had them* [sit-stand workstations] *so when one of us would stand up it would prompt the rest of us to. And we used to guilt each other a little bit into standing. It was like “You haven’t stood today what’s going on?” “Oh, yeah, alright, oh I’ve been a bit busy…” I suppose the mutual guilting into it worked quite well.* FG1 H8, female employee

The importance of social support for encouraging use of the intervention strategies was further highlighted through the contrasting perceptions from a couple of employees who were physically isolated from other study participants. They reported not using the desks as much as they would have liked.

*I didn’t have anyone else so there wasn’t quite that public knowledge on my level that I’m part of a study. There was a bit more self-conscious to actually like, stand up with my desk. I never did the standing up in meetings because I totally was self-conscious about it. There’s two people in our team meetings that would do that but that’s because they have an injury so I was actually thinking, “I can’t stand up because I don’t have that excuse, I don’t have any injury”* FG1 H14, female employee

Support from team leaders during the intervention was important in increasing the acceptability of the intervention and shifting organisational norms. Participants at most sites felt that they were supported by their team leaders and this made them feel more comfortable and confident taking up the intervention strategies.

*As soon as managers say, “If you want to stand, feel free to,” you can guarantee it there’ll be people immediately that will stand because managers have given them that permission to do it and therefore they’ve got the permission from everyone else to do it.* H3, female employee

#### Intervention effects on non-participants

As only selected teams within each worksite were invited to participate in the SUV trial, and some team members were ineligible, or chose not to participate, participants were often sitting adjacent to non-participants without sit-stand workstations. In some situations there were issues raised about audio and visual privacy, including increased noise when participants stood up or concerns about participants looking over them.

*They didn’t really look at where people were sitting before they put the stand-up desks in, and my friend just couldn’t use hers at all because where she was sitting there were people around her who were more sensitive to hearing her voice when she was standing up.* C9, female employee

Some team leaders managed this issue by moving employees or asking participants to lower their voice. In most sites, concerns about audio privacy became less of an issue over time. A few of the team leaders noted that noise was an unavoidable feature of open plan offices and such complaints were not solely attributable to the intervention.

*I just think you’ve got some loud talkers and you’ve got some quiet talkers. And being a team leader I had to move people because they didn’t want to sit next to the loud talker. This was before they even had the stand up desks.* J8, male team leader

#### Organisational support post-intervention

There was general uncertainty about the long-term commitment to the intervention messages from upper levels of management, particularly as the majority of sit-stand workstations had been removed after the study concluded. Participants reported having to *“jump through hoops”* to keep the desks, and that sit-stand workstations were only available to those who had been able to provide evidence from a medical practitioner that it was necessary for their health (see Table 3). This had created dissatisfaction and frustration amongst participants who wanted to—but were unable to—keep the desks and tension with the employees that were able to retain them.

*If the organisation was willing to just give them [sit-stand workstations] to every single person without a fight or without any qualms then I would be thinking that they're treating their staff as if they're important but at the moment they're still not really doing that. You've got to, you know, put in all your med certs [medical certificates of need] and you've got to have a reason why.* M8, female employee

Towards the end of the trial, non-participants had also started to express interest in obtaining a sit-stand workstation.

*When it* [the intervention] *was in its latter stages, people were saying, “how can I get one of those? How can I stand up?” Even though people weren't participating, they were seeing it and they must have been thinking about how it could be of some benefit to themselves.* A1, male team leader

### Processes of behavioural change

#### Sit-stand workstations as the key facilitator of behavioural change

While the SUV intervention was multi-component in nature, the sit-stand workstation was perceived to be the key driver of behavioural change and the principal element of the intervention. This likely reflected participants’ roles as predominately desk-based, with few job tasks able to be performed away from their workstations. The sit-stand workstation was perceived to be a more effective tool for reducing sitting than previously trialled strategies within the organisation, such as computer prompts.

*You need to have the physical ability to do it… I've had back issues in the past and I know I shouldn't sit for long. So before I had the Ergotron theoretically the Work Rate* [computer software] *would remind me to get up and walk around but if I'm in the middle of something I'll just, you know, skip it and keep working. Whereas I had a reminder… I would just, you know, lift the whole Ergotron up and keep working, no drama and then I'm standing.* M5, female team leader

After the desks were removed, participants reported it was difficult to increase their standing time in the context of predominately desk-based tasks.

*When you don’t have the desk I found it really hard to stand and work. Because everything like computer and the keyboard and the writing pads are always on the desk, so if you need to use any of those, you can’t really stand up and do it.* C2, male employee

The desk was also perceived to assist with normalising standing within the workplace. Without the desks, participants no longer felt they had a reason for standing that was justifiable to their colleagues.

*Now you stand up and you probably feel a little bit self-conscious because people are going “who’s that weirdo, what’s she doing –”, whereas if you’ve got the desk then people can see, “oh she’s got her desk up that’s why she’s standing.”* FG1 H14, female employee

#### Diversity in use and engagement with intervention strategies around ‘stand up, sit less and move more’

There was wide variety in participants’ reports of the ease and extent to which they were able to reduce their workplace sitting during the study. While some participants reported being highly motivated and driven to stand from the outset of the intervention, others perceived that they hadn’t changed as much as they had initially intended, noting the difficulty of shifting engrained habits of prolonged sitting or external factors (such as demanding caring responsibilities outside of work) that had limited their ability to engage with the intervention.

*I didn’t really use any prompts I think, I was already keen on standing so, you know, I just did it.* C5, male team leader

*I guess it's like an exercise or going for a run or a walk or something. When you're doing it it's feeling really good and you think, "I should do this all the time," but then somehow you don't do it. I think it's a bit like that. If it's there* [the sit-stand workstation] *I think that it will be utilised, but maybe not as often as it should be.* A3, female employee

Many spoke about the importance of self-motivation for successful behavioural change.

*the people that are motivated and are happier tend to adapt better to the stand up/sit down and are more able to follow and get the most out of the health benefits. The people that are less motivated, and are less happy, don't.* J7, female employee

Participants reported using a variety of strategies to increase their standing and moving time in the office. Commonly reported prompts used in conjunction with the workstation were time-based (e.g. use the workstation in the standing position during the morning) and task-based (e.g. stand while performing particular job tasks).

*The one that really stuck with me was at the end of the day to put the desk in the ‘up’ position. So I just came in to start the day with it ready to go.* FG1 H6, female team leader

*I tried to base it around the type of work… so at the end of that process you might say to yourself, “well I’ve been sitting down for a while so I can stand up now”* A6, male employee

Strategies most commonly used by participants to move more included: more frequent trips to the kitchen, bathroom, or to use the printer. A few reported going for walks during breaks. Participants also perceived that using their sit-stand workstation in the standing position made it easier to move more, as the transition to walking was easier from standing than from sitting.

*I felt like it* [standing] *was easy as a starting block. If I’m doing something and I have to go and see somebody it’s just easier to just walk instead of, ooh I have to get up like this and go.* FG1 H14, female employee

#### Health coaching and behavioural change

One-on-one health coaching was provided to all participants with the aim of supporting behavioural change. However, there were differing perspectives on its effectiveness (see Table 3) – those who considered themselves self-motivated did not perceive it to be very helpful.

*I didn’t find them useful, the strategies, because I was already doing it. I was self-motivated. I was very enthusiastic. I answered all my own questions. I just thought, great for people that needed it but I didn’t actually need it. I found it really pointless.* H3, female employee

Most frequently, participants saw the health coaching as a useful prompt or reminder by making them accountable to one of the study staff. One participant noted that the *“value was in the contact”* (K4, male employee) – having someone checking in to see how things were going. A few suggested that it would have been helpful for this check-in to have continued beyond the initial 3 month intervention period to support the sustainability of new habits.

*You know there’s going to be a contact in a month or two, you know you've got to be able to provide some feedback, so you've got to do the stuff in the meantime. You can't just not do anything. So probably one or two, even after the three months. Not to go through, "Look are you doing this, this, this, and this?" But just around saying, “How are you going? What do you think is working, what’s not working?”* K4, male employee

## Discussion

These qualitative findings provide insights into the experiences of office workers during a 12 month intervention to reduce workplace sitting, including perceived barriers and facilitators to behavioural change, and effects of the intervention on workplace culture and work performance. They have relevance for research and practice by highlighting contextual factors that may need to be considered when implementing interventions, in order to increase the likelihood of reducing and breaking up sitting time.

Overall, the majority of participants reported positive experiences with the intervention and were interested in retaining the sit-stand workstations at the conclusion of the 12 month period. While there were a small minority of participants who reported negative physical effects (e.g. musculoskeletal problems) during the trial as whole [17], participants in these qualitative interviews perceived that the increased workplace standing time had had positive impacts on alertness, concentration and energy, and for some, had relieved their musculoskeletal complaints. These observations are consistent with a growing body of research suggesting that workers perceive a range of health and well-being benefits from using a sit-stand workstation [15, 16, 20]. However, despite the potential for sit-stand workstations to be an effective health promoting strategy, previous studies have suggested that the workstations are viewed as an aid for addressing pre-existing musculoskeletal conditions, rather than for preventive health [21, 22]. This mindset is likely to continue to be perpetuated while sit-stand workstations are only provided selectively, rather than universally to all employees.

When prompted about their perceived productivity during the trial, participants generally reported that the use of the sit-stand workstations had had no noticeable impact on their performance. Some reported feeling that increased standing facilitated improved communication and interaction with co-workers, which may be beneficial to team performance. However, there appeared to be certain job tasks that were more difficult to perform standing with this particular workstation, leaving some participants sitting more than desired. A number of studies have now reported that workers perceive either a negligible or positive impact on self-reported work performance when using sit-stand workstations for short periods of time [13, 14, 16, 23–25]. In addition, a recent study evaluating the impact of sit-stand workstations on work performance amongst call centre workers found no significant difference in a number of objective measures of productivity, including call handling time, attendance and sick leave [9]. Longer term studies are required to assess productivity effects for organisations at a macro level; however, it is a positive finding that in the short-medium term, sit-stand workstations appear to have negligible negative impact on work performance.

The intervention encompassed strategies acting at the individual, organisational and environmental-levels. There appeared to be variation in the strategies that participants found to be useful. Overall, the sit-stand workstation was reported to be the main driver of behavioural change, with most reporting that they were unable to reduce their sitting time after the workstations were removed. This is indicative of the job roles of participants, which included predominately desk and phone-based tasks. From a whole of workday perspective, sit-stand workstations will have greater potential to facilitate large reductions in sitting time relative to other strategies that were promoted, such as standing in meetings. However, consistent with an ecological model of sedentary behaviour [11], individual and social factors interacted with the environmental modification provided by the sit-stand workstation to influence behaviour. In particular, the social support provided by other participants (e.g. through seeing others stand up with the workstation) and team leaders (who provided permission and support for change) were perceived to be important facilitators. In contrast, those who were physically isolated from other users of sit-stand workstations felt more self-conscious using the workstations. Others [25] have reported similar findings, suggesting the importance of the social environment for facilitating, or impeding, reductions in workplace sitting time.

Organisational cultural norms around “appropriate” workplace behaviour have previously been found to be barriers to behavioural change when attempting to shift engrained patterns of workplace sitting [21]. Some participants reported that the sit-stand workstation itself appeared to influence social norms by facilitating a cultural shift in the acceptability of standing and moving more within the workplace. However, where participants were isolated from other study participants, cultural shifts were less apparent. In some sites, tensions between participants and non-participants over issues such as audio and visual privacy led to some not fully utilising the workstations. Some of these issues reflect the limitations of a research trial as not all employees were eligible or consented to participate. However, these findings may have implications for worksites considering a selective roll-out of sit-stand workstations. Programs that aim to engage entire worksites, and consider workspace design and layout, may be more successful in shifting workplace culture and organisational norms towards acceptance of reducing workplace sitting.

Despite an overall positive view of sit-stand workstations in general, most participants had some negative feedback about the design of the particular model trialled (Ergotron WorkFit-S), including the size of the work surface and workstation stability. Previous studies using the same or similar models have reported comparable feedback [13, 15, 16, 25], suggesting that workstation design is an important consideration. Since the study commenced in 2012, a number of other sit-stand workstation models have come on to the market, many of which provide larger, more stable work surfaces, which may address these shortcomings.

Many participants wished to retain the workstations at the conclusion of the trial despite these concerns about the design, suggesting that interest in sit-stand workstations can be sustained over the medium-long term. However, it is worth noting that, on average, participants reduced the amount of time spent standing as the study progressed [17]. There is a need for additional research to identify the determinants of sustained behavioural change over time, particularly at the organisational and environmental level.

### Lessons learned from the SUV trial and recommendations for future workplace sitting programs/interventions

The findings from this study have implications for researchers designing interventions and for organisations seeking to promote more activity-friendly environments for their employees. Table 4 summarises the lessons learned from the SUV intervention and key recommendations for future workplace programs.

**Table 4:** Implementing strategies to reduce workplace sitting: lessons from the SUV trial and recommendations for research, policy and practice

Lessons learned	Recommendations
• Awareness of current activity levels (i.e. time spent sitting and moving) may be important for behavioural change.	• Assess and provide feedback on employees’ behaviour, preferably with objective measures. • If resources do not facilitate objective measurement, a questionnaire can provide relevant insights.
• Sit-stand workstations are integral to achieving large reductions in workplace sitting time for those with largely desk-based roles. Participants in this study reported having limited opportunities to stand once the workstations were removed.	• Provide employees with access to sit-stand workstations where organisational resources permit. • Attempt to replace fixed height workstations with sit-stand workstations (or static standing workstation options) during scheduled office furniture upgrades. • Provide opportunities for job tasks to be performed at alternative work points (e.g. communal standing or sit-stand workstations) to encourage greater movement throughout the day.
• The design of sit-stand workstations can be a barrier to use. Stability and size of the work surfaces are important features.	• When selecting sit-stand workstations for purchase consider: - Ease of movement (manual vs electric adjustment; speed and noise of movement) - Ergonomic and occupational health and safety (OHS) requirements (compliance with standard height range, height indicators to facilitate use at appropriate height, addressing any pinch points associated with moving elements). - Suitability of work surface size and monitor arrangement for predominant job tasks)
• Installing sit-stand workstations in open plan environments can have implications for audio and visual privacy, particularly when the provision of workstations is not universal.	• Create supportive social and environmental conditions to support sit-stand workstations. For example, higher partitions, separate quiet spaces for phone calls, reorienting of desks or relocation of workers. • Provide and encourage use of alternative work points with audio and visual privacy to support tasks, such as phone calls. • Managers/team leaders should monitor interactions between workers and provide advice/conflict resolution as needed.
• A whole of organisation approach to promoting sit less, move more strategies is important, including support from middle and senior levels of management as well as peer support.	• Managers/team leaders should lead by example and support and encourage sit less strategies. For example, providing permission for employees to stand in meetings. • Co-workers play an important role in prompting and supporting positive behaviour change.
• Existing preconceptions around sit-stand workstations and their purpose may be a barrier to their use. For example, workstations traditionally only being provided to those with musculoskeletal issues.	• Review and, where appropriate, update and promote policies around the provision and use of sit-stand workstations. • Ensure that key business stakeholders, including OHS representatives, are included in process of on-boarding sit-stand workstations to increase their relevance. • Consider piloting sit-stand workstations to increase positive perceptions and knowledge prior to full roll out.
• Ongoing support and encouragement is important for the creation of new habits relating to sitting less and moving more. For some employees this may be required for longer than 3 months.	• Discuss benefits and challenges to reducing sitting through organisation social media platforms and intranets. • Regular competitions or events to promote sitting less may assist to reinvigorate strategies. • Use signage to provide behavioural prompts to reduce workplace sitting.

This study adds to and extends the growing literature evaluating participants’ perspectives of sit-stand workstations in office workplaces [13–15, 23, 26]. Similar themes were identified to those in previous research relating to perceived barriers and facilitators to using sit-stand workstations [13, 15]. In addition to evidence of their efficacy for reducing workplace sitting time [17], this study suggests that sit-stand workstations are acceptable and feasible to office-based employees across the medium-long term, and are perceived to have positive physical and mental impacts. However, it is acknowledged that the cost implications of purchasing sit-stand workstations for all employees may be a barrier for some organisations. A comprehensive cost-effectiveness analysis of the SUV intervention is currently in progress, which will provide insight into the economic credentials of the intervention, including the costs of the workstations.

Key strengths of the present study include the 12 month duration of the intervention, medium to long-term follow up and the ecological validity, featuring office workers across multiple geographically separate worksites. It is worth noting, however, that this qualitative study was an additional voluntary component of the SUV trial, occurring after the intervention had concluded. While participants did appear to be broadly representative of intervention participants in terms of socio-demographics, workplace sitting reductions were higher among these participants than intervention participants as a whole. Participants who volunteered for the qualitative component may have been more engaged than those who did not—potentially biased towards those who wished to retain the sit-stand workstations—which could mean these findings present an overly favourable impression of the intervention. However, it is worth noting that this study found a range of favourable and unfavourable perspectives. This study also did not approach workers who initially consented to the trial but withdrew prior to the study completion, who may have had more adverse experiences. The use of focus groups at only one of the participating sites is also a limitation, however arguably this is offset by the insights gained into group-level dynamics during the intervention. Finally, the interviews were conducted between one and 4 months after the intervention had concluded, which may have affected participant recall, and only at one time point, limiting understanding of whether participants’ experience of the intervention changed over time. Future studies should consider conducting qualitative interviews at both short and long-term follow-up periods to gain these perspectives.

## Conclusions

These findings are supportive of the notion that sit-stand workstations are an effective and acceptable method for reducing sitting time in office workers. However, to support their use, best practice workplace initiatives should be multi-component in nature and address the individual, social and environmental-related influences that may act as barriers to effective uptake. The findings from this study and suggested recommendations may be informative for organisations considering approaches for reducing workplace sitting.

## Additional file

Additional file 1: Questions covered during the focus group discussions. (DOCX 15 kb)

## Abbreviations

BMI: Body mass index
FTE: Full-time equivalent
SUV: Stand Up Victoria

## References

[1] Healy G, Lawler S, Thorp A, Neuhaus M, Robson E, Owen N, et al. Reducing prolonged sitting in the workplace (an evidence review: full report). Melbourne, Australia: Victorian Health Promotion Foundation; 2012.

[2] Chau JY, Grunseit AC, Chey T, Stamatakis E, Brown WJ, Matthews CE, et al. Daily sitting time and all-cause mortality: a meta-analysis. PLoS One. 2013;8:e80000. 10.1371/journal.pone.0080000 PMC3827429 24236168

[3] Biswas A, Oh PI, Faulkner GE, Bajaj RR, Silver MA, Mitchell MS, et al. Sedentary time and its association with risk for disease incidence, mortality, and hospitalization in adults: a systematic review and meta-analysis. Ann Intern med. 2015;162:123–32. 10.7326/M14-1651 25599350

[4] de Rezende LF, Rodrigues Lopes M, Rey-Lopez JP, Matsudo VK, Luiz OC (2014). Sedentary behavior and health outcomes: an overview of systematic reviews. PLoS One.

[5] Chu AH, Ng SH, Tan CS, Win AM, Koh D, Muller-Riemenschneider F (2016). A systematic review and meta-analysis of workplace intervention strategies to reduce sedentary time in white-collar workers. Obes rev.

[6] Neuhaus M, Eakin EG, Straker L, Owen N, Dunstan DW, Reid N, et al. Reducing occupational sedentary time: a systematic review and meta-analysis of evidence on activity-permissive workstations. Obes rev. 2014;15:822–38. 10.1111/obr.12201 25040784

[7] Shrestha N, Kukkonen-Harjula KT, Verbeek JH, Ijaz S, Hermans V, Bhaumik S (2016). Workplace interventions for reducing sitting at work. Cochrane Database Syst rev.

[8] Healy GN, Eakin EG, Lamontagne AD, Owen N, Winkler EA, Wiesner G, et al. Reducing sitting time in office workers: short-term efficacy of a multicomponent intervention. Prev med. 2013;57:43–8. 10.1016/j.ypmed.2013.04.004 23597658

[9] Chau JY, Sukala W, Fedel K, Do A, Engelen L, Kingham M, et al. More standing and just as productive: effects of a sit-stand desk intervention on call center workers' sitting, standing, and productivity at work in the opt to stand pilot study. Prev Med Rep. 2016;3:68–74. 10.1016/j.pmedr.2015.12.003 PMC4733097 26844191

[10] Tobin R, Leavy J, Jancey J (2016). Uprising: an examination of sit-stand workstations, mental health and work ability in sedentary office workers, in Western Australia. Work.

[11] Owen N, Sugiyama T, Eakin EE, Gardiner PA, Tremblay MS, Sallis JF (2011). Adults' sedentary behavior determinants and interventions. Am J Prev med.

[12] Neuhaus M, Healy GN, Dunstan DW, Owen N, Eakin EG (2014). Workplace sitting and height-adjustable workstations: a randomized controlled trial. Am J Prev med.

[13] Chau JY, Daley M, Srinivasan A, Dunn S, Bauman AE, van der Ploeg HP (2014). Desk-based workers' perspectives on using sit-stand workstations: a qualitative analysis of the stand@work study. BMC Public Health.

[14] Grunseit AC, Chau JY, van der Ploeg HP, Bauman A (2013). "thinking on your feet": a qualitative evaluation of sit-stand desks in an Australian workplace. BMC Public Health.

[15] Leavy J, Jancey J (2016). Stand by me: qualitative insights into the ease of use of adjustable workstations. AIMS Public Health.

[16] Dutta N, Walton T, Pereira MA (2015). Experience of switching from a traditional sitting workstation to a sit-stand workstation in sedentary office workers. Work.

[17] Healy GN, Eakin EG, Owen N, LaMontagne AD, Moodie M, Winkler EA, et al. A cluster RCT to reduce office workers' sitting time: impact on activity outcomes. Med Sci Sports Exerc. 2016;48:1787–97. 10.1249/MSS.0000000000000972 27526175

[18] Dunstan DW, Wiesner G, Eakin EG, Neuhaus M, Owen N, LaMontagne AD, et al. Reducing office workers' sitting time: rationale and study design for the stand up Victoria cluster randomized trial. BMC Public Health. 2013;13:1057. 10.1186/1471-2458-13-1057 PMC3828481 24209423

[19] Neuhaus M, Healy GN, Fjeldsoe BS, Lawler S, Owen N, Dunstan DW, et al. Iterative development of stand up Australia: a multi-component intervention to reduce workplace sitting. Int J Behav Nutr Phys act. 2014;11:21. 10.1186/1479-5868-11-21 PMC3936706 24559162

[20] Thorp AA, Kingwell BA, Owen N, Dunstan DW (2014). Breaking up workplace sitting time with intermittent standing bouts improves fatigue and musculoskeletal discomfort in overweight/obese office workers. Occup Environ med.

[21] Hadgraft NT, Brakenridge CL, LaMontagne AD, Fjeldsoe BS, Lynch BM, Dunstan DW, et al. Feasibility and acceptability of reducing workplace sitting time: a qualitative study with Australian office workers. BMC Public Health. 2016;16:933. 10.1186/s12889-016-3611-y PMC5011963 27595754

[22] Gilson ND, Suppini A, Ryde GC, Brown HE, Brown WJ (2012). Does the use of standing 'hot' desks change sedentary work time in an open plan office?. Prev med.

[23] Dutta N, Koepp GA, Stovitz SD, Levine JA, Pereira MA (2014). Using sit-stand workstations to decrease sedentary time in office workers: a randomized crossover trial. Int J Environ res Public Health.

[24] Pronk NP, Katz AS, Lowry M, Payfer JR (2012). Reducing occupational sitting time and improving worker health: the take-a-stand project, 2011. Prev Chronic Dis.

[25] Graves LEF, Murphy RC, Shepherd SO, Cabot J, Hopkins ND (2015). Evaluation of sit-stand workstations in an office setting: a randomised controlled trial. BMC Public Health.

[26] Alkhajah TA, Reeves MM, Eakin EG, Winkler EA, Owen N, Healy GN (2012). Sit-stand workstations: a pilot intervention to reduce office sitting time. Am J Prev med.

