# Supplementary material for: Reducing occupational sitting: Workers’ perspectives on participation in a multi-component intervention
Source: Int J Behav Nutr Phys Act. 2017 May 30;14:73. doi: 10.1186/s12966-017-0530-y (PMC5450410; doi:10.1186/s12966-017-0530-y)
Supplement: Additional file 1: — Questions covered during the focus group discussions. (DOCX 15 kb) [file 12966_2017_530_MOESM1_ESM.docx]

**Additional File 1:**

**Questions covered during the focus group discussions**

| *Theme: Global Satisfaction*   1. **How was your overall experience with the Stand Up Victoria study?**    - How satisfied were you with your experience?    - What were some of the positives about the experience?    - What do you see as the advantage/s of standing more at work?    - Do you feel that the movement improved your wellbeing/comfort? How?    - What could be improved upon in future projects that are introducing sit/stand workstations? (Be sure to let participants know that we have no commercial interest in the workstations)    - Do you feel that the movement within the workplace negatively impacted on the way in which you worked? How?    - Do you feel like you could control the workstation with ease? i.e., do you think you could make the change from standing to sitting easily? If not, why?    - Did you feel that you were provided with the right knowledge to allow you to stand up, sit less, and move more within your workplace?    - Would you recommend sit-stand works stations to others?  Why/Why not?   *Theme: Motivation and sustainability*   1. **If each of you could reflect on your own individual strategies:**    - Which ones motivated you the most to change your working position? Why do you think that was?    - Where there any strategies that you tried that didn’t work? Why do you think that was?    - If changes were made, what is it going to take for these changes to become sustainable in your group in the long-term? 2. **Now that the study is over and your desks have been removed, are you still trying to follow the objectives of the study – standing up, sitting less, moving more?** 3. **How suitable do you think your workplace is for sit/stand workstations? If not, what needs to change to make it suitable?** 4. **Could you see your workplace taking on any other changes now that the study is complete?**   *Theme: Workplace Culture*   1. **To what extent do you feel the workplace ‘culture’ has changed to support the Stand Up Sit Less Move More messages?**   *Prompts*   - - Did the opportunity to have a sit-stand workstation make you feel more valued as employees?   - Did the sit-stand workstation make you feel more in control of your workspace?   - Did you feel you had the support of your team leader/upper management to make these changes at your workstation? Why, why not?   - Where you conscious of your perceived productivity as a result of using the workstation?   - How did the sit-stand workstation impact on your sense of privacy? (audio vs. visual)   - Did you feel that the sit-stand workstations impacted on the sense of audio/ visual privacy of others around you? If yes, how so?   - Where there others in close proximity to you that had sit-stand workstations?     - If no, did you feel comfortable changing your behaviour in such an isolated area?     - If yes, did being in close proximity with others with workstations have an impact on your behaviour? Were you encouraged/discouraged from changing your behaviour as a result?   *Theme: Productivity*   1. **What did you think about the impact of the workstations on your productivity, in terms of:**    - Communication (between each other and clients)    - Collaboration    - Task completion/ work flow |
| --- |
